# Breastfeeding Promotion in Maternity Wards From a Socioeconomic View

**DOI:** 10.1002/fsn3.71045

**Published:** 2025-10-15

**Authors:** Christina Kürten, Nele Hockamp, Kathrin Sinningen, Erika Sievers, Thomas Lücke, Mathilde Kersting

**Affiliations:** ^1^ Research Department of Child Nutrition, University Hospital of Pediatrics and Adolescent Medicine, St. Josef Hospital Ruhr‐University Bochum Bochum Germany; ^2^ Freelance Researcher Haale Germany

**Keywords:** breastfeeding, breastfeeding promotion, maternity hospitals, public health, social inequalities, socioeconomic status, WHO/UNICEF ten steps

## Abstract

Social disparities in breastfeeding may result in inequalities of maternal and child health‐related future perspectives. In‐hospital breastfeeding promotion, information, and support impact short‐ and long‐term breastfeeding practices. This study, which was performed in 2021/2022 in the context of the COVID‐19 pandemic, examined prepandemic interrelations between in‐hospital breastfeeding promotion, information, and support in the federal state North Rhine‐Westphalia (NRW), Germany, and the socioeconomic structure of the hospitals' catchment areas. Breastfeeding promotion, information, and support were operationalized using a Breastfeeding Promotion Index (BPI) based on the “Ten Steps to Successful Breastfeeding” postulated by the World Health Organisation (WHO) and the United Nations Children's Fund (UNICEF) and adapted for Germany by the National Breastfeeding Committee. The socioeconomic structure of the districts hosting the hospitals was assessed by the German Index of Socioeconomic Deprivation (GISD), which was developed by the Robert Koch Institute and is established for Public Health issues. This was complemented by the head physicians' estimations of the predominant socioeconomic status in their hospitals' catchment areas. Statistical analyses were performed using the non‐parametric Jonckheere‐Terpstra's test. For correlation analyses, Spearman's Rho and Kendall tau c were used. A low socioeconomic status was associated with a lower BPI and, vice versa, a high socioeconomic status with a higher BPI. These relationships were stronger when using the head physicians' estimations than the GISD. Higher breastfeeding rates at discharge as estimated by the maternity ward staff were favored by a higher BPI and a higher regional socioeconomic status. These data suggest that hospitals may be able to assess their need for intensified breastfeeding promotion and support strategies by identifying the predominant socioeconomic status of their catchment areas. The results also point to the “disproportionate care law” which may still apply in the well‐established German healthcare system.

## Graphical abstract

Head physicians of maternity hospitals are able to accurately estimate the socioeconomic structure of their hospitals' catchment areas, which can serve as a measure for the necessity of intensified breastfeeding promotion activities. Breastfeeding at discharge is more likely in hospitals with a socially advantaged catchment area.

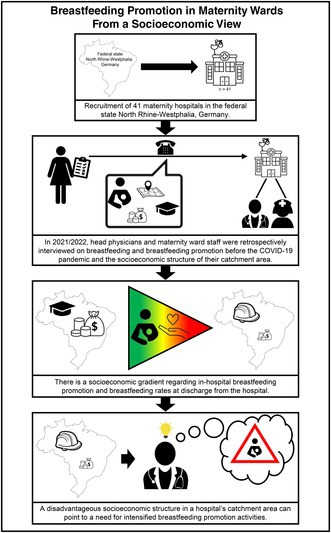

## 1 Introduction

Breastfeeding is the gold standard of infant nutrition in the first months of life. Due to its health benefits for mothers and children (Masi and Stewart 2024), it has increasingly raised public health interest. WHO and UNICEF postulated the “Ten Steps to Successful Breastfeeding” (WHO 1989; WHO and UNICEF 2018), hereafter referred to as the “Ten Steps”, which should be implemented by hospitals providing maternity services. These recommendations have been adapted for Germany by the National Breastfeeding Committee (German Federal Institute for Risk Assessment 2007). Breastfeeding promotion, information, and support in hospitals‐for example, by implementing the Ten Steps‐can have a beneficial effect on in‐hospital and subsequent outpatient breastfeeding habits (Pérez‐Escamilla et al. 2016; Pérez‐Escamilla et al. 2023; Finnie et al. 2020; Hockamp et al. 2022). The implementation of the Ten Steps can be assessed by the Breastfeeding Promotion Index (BPI) that enables the differentiation of a low, medium, and high implementation level (Dulon et al. 2003; Hockamp et al. 2022). The Ten Steps and the BPI contain measures of breastfeeding promotion and information, as well as support (WHO and UNICEF 2018). Therefore, the term “breastfeeding promotion” is used hereafter in a summarizing sense of these aspects.

Over the last decades, breastfeeding rates have risen in high‐income countries (Lanting et al. 2005; Neves et al. 2021; Vaz et al. 2021), also in Germany (Brettschneider et al. 2018; Dulon et al. 2001; Hockamp et al. 2021). Contrasting high breastfeeding initiation rates, the duration of (exclusive) breastfeeding often remains below the recommendations (Vaz et al. 2021; Brettschneider et al. 2018; Bürger et al. 2022; Victora et al. 2016; Weissenborn et al. 2016). Social disparities exist regarding breastfeeding habits: mothers of a low social status have an increased risk of suboptimal breastfeeding (Hunt et al. 2022; Foster et al. 2023; Lange et al. 2007). Especially, the educational level of the mothers is a strong predictor of breastfeeding behavior (van Neste et al. 2025). For instance, a German prospective study conducted by Logan et al. revealed declining early cessation rates in 2012/2013 compared to 2000/2001. However, this trend was restricted to mothers with a high educational level, leading to an enlargement of social disparities in breastfeeding (Logan et al. 2016). A lack of targeted strategies for breastfeeding promotion in vulnerable groups in US maternity institutions contrasts with the distinct awareness regarding social and racial disparities (Gonzalez‐Nahm and Benjamin‐Neelon 2023). The “cycle of malnutrition” is of high relevance in low‐ and middle‐income countries. Due to structural problems and racism, relevant transgenerational inequities in food safety can be found even within high‐income countries such as the US (Tomori 2023). The inverse/disproportionate care law, which describes that socioeconomically weak populations receive relatively insufficient health support compared to their greater need (Tudor Hart 1971; Cookson et al. 2021), is of high importance in this context. Due to the potential health benefits of breastfeeding for mothers and children, the reduction of social disparities in breastfeeding is an important public health issue.

Most studies focus on mother‐infant characteristics when investigating breastfeeding promotion strategies. However, there is little information on breastfeeding promotion and support in relation to breastfeeding practices in the context of the maternity hospitals' socioeconomic environments. The regional socioeconomic structure can be assessed by using the German Index of Socioeconomic Deprivation (GISD; Michalski et al. 2022).

The objectives of this exploratory study, based on information given on the prepandemic conditions, were:

1. To compare the head physicians' estimations of the predominant socioeconomic status in the hospitals' catchment areas to the GISD of the corresponding districts.
2. To examine the relations between in‐hospital breastfeeding promotion and the socioeconomic structure of the hospitals' catchment areas.
3. To examine the relations between in‐hospital breastfeeding promotion, the socioeconomic structure of the hospitals' catchment areas, and estimated breastfeeding rates at discharge.

## 2 Methods

### 2.1 Study Design

The SINA‐study (abbreviation for Stillen in Nordrhein‐Westfalen (NRW); breastfeeding in North Rhine‐Westphalia) was conducted between October 2021 and March 2022 and thus in the context of the COVID‐19 pandemic. Data were collected in partially standardized telephone interviews. Both the head physicians and the maternity ward staff responsible for breastfeeding were interviewed. The interviews focused on the circumstances, challenges, strategies, and implications of breastfeeding promotion, information, and support containing a comparison of the situation before and during the COVID‐19 pandemic. They included questions regarding the Ten Steps (WHO and UNICEF 2018; German Federal Institute for Risk Assessment 2007). The questions were based on the SuSe (abbreviation for Stillen und Säuglingsernährung; breastfeeding and infant nutrition)—II study (Hockamp et al. 2021) and expanded to questions regarding the pandemic situation and vulnerable sociodemographic groups. This publication focuses on information on prepandemic conditions as they prevailed in 2019. For an overview of the results, see Research Department of Child Nutrition (2023). The data that support the findings of this study are available from the corresponding author upon reasonable request.

### 2.2 Study Sample

**FIGURE 1:**
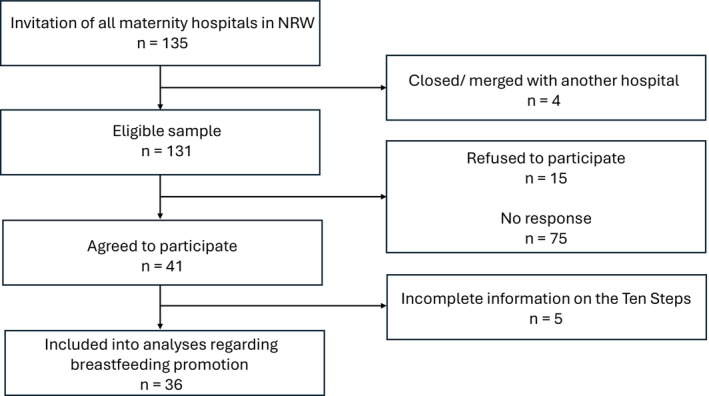
Flowchart of recruitment and participation.

Based on a German‐wide birth list (Milupa Nutricia GmbH 2021), a total of 135 maternity wards in NRW was identified and received written invitations to participate in the study. Four hospitals were excluded because of a merger with another hospital or closure of the department. Due to a low initial response rate, hospitals were additionally contacted by phone and received information via e‐mail and/or fax. A sample of 41 maternity hospitals, hereafter referred to as “hospitals,” finally participated in the study. Five hospitals were excluded from analyses regarding breastfeeding promotion because they provided incomplete or unclear information on the Ten Steps (Figure 1). Analyses not referring to the Ten Steps were performed on the complete sample of 41 hospitals.

### 2.3 Socioeconomic Structure

The regional socioeconomic structure was included in the analysis using the GISD of the districts or independent cities in which the hospitals were located for the prepandemic year 2019 (Michalski et al. 2024). The term “district” refers to an administrative level serving supra‐regional administrative tasks in Germany (Research Center Jülich GmbH n.d.). 107 of the 295 districts in Germany are “independent cities”: due to a high population count, these cities also form a district (Research Center Jülich GmbH n.d.). The GISD was developed and provided by the Robert Koch Institute (Kroll et al. 2017). It is a measure of the socioeconomic situation of the corresponding region's population compared to the German population in total and is used for Public Health‐related purposes. It contains nine indicators regarding the equally weighted categories education, profession, and income. Therefore, the GISD provides an analogy to the term “socioeconomic status” (SES), which refers to individual persons. It ranges from 0 (lowest deprivation) to 1 (highest deprivation). Categorization is conducted by forming quintiles: socioeconomic deprivation is labeled as “high” in the highest quintile, “low” in the lowest quintile, and “medium” in the three remaining quintiles (Michalski et al. 2022). Quintiles were formed within the study sample and included in the analysis as described above. Besides the GISD, the head physicians' estimations of the predominant socioeconomic status (low, medium, high) in the hospitals' catchment areas (containing, e.g., the unemployment rate and the proportion of single‐parent households) were considered. Hereafter, the term “hospital estimated socioeconomic status” (HESES) or “HESES group” refers to this estimation, while “GISD groups” refers to the abovementioned quintiles.

### 2.4 Assessment of Breastfeeding Promotion, Information and Support

The implementation of the Ten Steps, adapted to Germany (German Federal Institute for Risk Assessment 2007), was determined for each hospital. The total number of implemented steps was calculated for each hospital, which corresponds to the BPI. Subsequently, the hospitals were divided into three groups (low/medium/high BPI; hereafter referred to as “BPI groups”) using the 25th and 75th percentiles as cut‐offs.

Further information included was (1) the rate of any breastfeeding mothers at discharge, (2) the rate of exclusively breastfeeding mothers at discharge (both as estimated by maternity ward staff), and (3) the provision of support services for families with special needs post‐discharge. Referring to the WHO guidelines published in 2007 and 2021 (WHO 2007; WHO and UNICEF 2021), any breastfeeding means that the infant may receive other liquids/food in addition to breast milk, whereby exclusively breastfed infants do not receive any liquids or food other than breast milk.

### 2.5 Statistical Analysis

First, hospitals were divided into the three HESES groups and compared in terms of GISD and BPI as well as regarding the estimated breastfeeding rates and the provision of outpatient support services. The group comparison was repeated by using the GISD as the discriminating factor. Secondly, the BPI groups were compared regarding the corresponding regions' GISD and the estimated breastfeeding rates. Finally, group comparisons were supplemented by correlation analyses of the non‐categorized data.

Data corresponding to hospital groups are displayed as mean value ± standard deviation. These data are supplemented by median values when necessary. Due to the small sample size, only non‐parametric tests were used for further analysis. Differences between the groups were tested for significance using Jonckheere‐Terpstra's test after variances were tested for homogeneity performing Levene's test. Significance in Jonckheere‐Terpstra's test indicates a significant median difference between two or more groups containing a trend (Bewick et al. 2004). Correlation analyses were performed using Spearman's Rho and Kendall Tau c. The level of significance was set at *p* < 0.05 (2‐sided). Analyses were conducted using the IBM SPSS Statistics version 29.0.2.0 software package for Windows Version 2023 (IBM Corporation).

## 3 Results

### 3.1 Hospital Characteristics

The sample comprised 41 and 36 hospitals, respectively (Figure 1). The annual number of births in the current sample was higher (1619 ± 727 births) than the average number of births in all 135 maternity wards in NRW (1198 ± 636 births) in 2020. The study sample contains a larger proportion of hospitals with more than 1000 births (80.49% in the sample vs. 53.6% in the total of hospitals) and fewer hospitals with less than 500 (2.44% vs. 8.6%) and 500–1000 (17.07% vs. 37.7%) births (Milupa Nutricia GmbH 2021). This sample showed a slightly lower percentage of hospitals owned by non‐profit and private institutions and therefore a higher percentage of hospitals under public ownership compared to the maternity hospitals in NRW in total (German Federal Statistical Office 2022). A subgroup of 26 hospitals within the study sample was perinatal care centers. Their composition regarding the levels of perinatal care is quite similar to the total of hospitals in NRW. There is only a slight underrepresentation of level II centers in favor of the level I centers (Richter et al. 2022). For detailed hospital characteristics in comparison to the total of maternity wards in NRW, see Table A1.

### 3.2 Comparison of the Assessment Approaches of the Socioeconomic Structure

**FIGURE 2:**
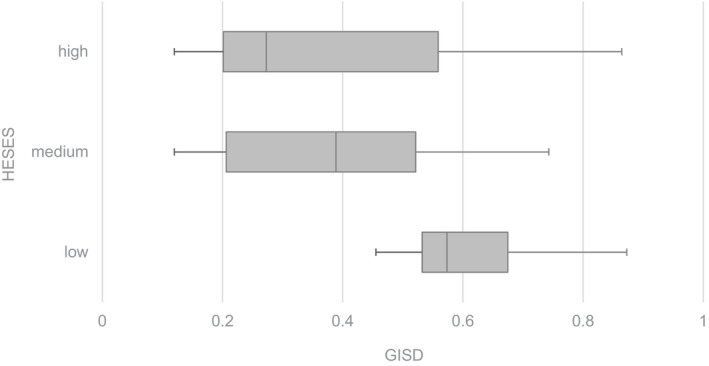
Boxplots showing the median, 25th percentile, 75th percentile, minimum, and maximum of the GISD (German Index of Socioeconomic Deprivation) within the HESES (hospital estimated socioeconomic status) groups. Jonckheere–Terpstra's test for HESES group comparison: *p* = 0.003.

The GISD of the districts and free cities where the hospitals were situated showed a huge variance. The mean GISD for the sample was 0.442 ± 0.209 (median 0.453; min. 0.120; max. 0.873). HESES was estimated as medium in the largest proportion (23 hospitals ≜ 56.1%) of hospitals (low: 10 hospitals ≜ 24.4%; high: 8 hospitals ≜ 19.5%). These groups differed significantly regarding the median GISD (Figure 2): The higher the HESES was, the lower the GISD was (*p* = 0.003): in the low HESES group, the median GISD was 0.574 and thus higher than in the medium (0.389) and high (0.273) HESES group.

### 3.3 Breastfeeding Promotion, Information and Support in Hospitals

On average, 6.25 ± 1.42 (median 6.0; min. 3, max. 8) of the adapted Ten Steps as given in Table 1 were implemented. Referring to the 25th and 75th percentiles, hospitals with a low BPI implemented five steps maximum, a medium BPI corresponds to six or seven implemented steps, and a high BPI equated to eight implemented steps minimum. The three BPI groups comprised of *n* = 9 hospitals (low BPI), *n* = 19 hospitals (medium BPI), and *n* = 8 hospitals (high BPI). The median number of implemented recommendations differed significantly between the three groups (low BPI: 4.0; medium BPI: 6.0; high BPI: 8.0; *p* < 0.001). Detailed information on the implementation of the individual steps can be found in Table 1.

**TABLE 1:** Ten steps to successful breastfeeding.

Step	Recommendations	Implemented in % of hospitals			
		Total	Low BPI	Medium BPI	High BPI
1	Have a written breastfeeding policy and a breastfeeding coordinator^a^				
	Step 1b: Have a written infant feeding policy that is routinely communicated to staff and parents^b^				
	*There is a breastfeeding coordinator* ^c^	66.7%	33.3%	73.7%	87.5%
2	Train all health care staff in theory and practice to implement the guidelines for the promotion of breastfeeding^a^				
	Ensure that staff have sufficient knowledge, competence and skills to support breastfeeding^b^				
	*Head physicians report (a) the whole team or (b) physicians, midwifes and nurses to receive training on breastfeeding* ^c^	58.3%	33.3%	57.9%	87.5%
3	Mothers receive information on breastfeeding before birth, including written information provided by the National Breastfeeding Committee^a^				
	Discuss the importance and management of breastfeeding with pregnant women and their families^b^				
	*Mothers receive information on breastfeeding before birth, in personal contact and in written form* ^c^	44.4%	22.2%	31.6%	100.0%
4	Early initiation—if possible within the first hour of life.^a^				
	Facilitate immediate and uninterrupted skin‐to‐skin contact and support mothers to initiate breastfeeding as soon as possible after birth^b^				
	*Breastfeeding is initiated within the first hour of life after a Caesarean section and after a spontaneous birth* ^c^	27.8%	0.0%	42.1%	25.0%
5	Show mothers how to breastfeed their child and to maintain milk production, even if separated from their child^a^				
	Support mothers to initiate and maintain breastfeeding and manage common difficulties^b^				
	*Practical support on breastfeeding is provided by default* ^c^	77.8%	88.9%	73.7%	75.0%
6	Additional feeding if medically indicated^a^				
	Do not provide breastfed newborns any food or fluids other than breast milk, unless medically indicated^b^				
	*Physicians (and mothers) decide about the mandatorily medical indication for additional feeding* ^c^	19.4%	11.1%	15.8%	37.5%
7	Support mother–child unity—enable 24‐h rooming‐in^a^				
	Enable mothers and their infants to remain together and to practice rooming‐in 24 h a day^b^				
	*24 h‐rooming‐in is provided* ^c^	91.7%	77.8%	94.7%	100.0%
8	Breastfeeding on demand—breastfeeding should be made possible and encouraged as needed^a^				
	Support mothers to recognize and respond to their infants' cues for feeding^b^				
	*Breastfeeding is enabled and supported without a fix schedule* ^c^	100.0%	100.0%	100.0%	100.0%
9	Alternative feeding methods (e.g., cup, finger and spoon feeding) should be shown^a^				
	Counsel mothers on the use and risks of feeding bottles, teats and pacifiers^b^				
	*Alternative feeding methods are shown if additional feeding is required* ^c^	80.6%	33.3%	94.7%	100.0%
10	Encourage the establishment of breastfeeding groups and provide mothers contact information at discharge^a^				
	Coordinate discharge so that parents and their infants have timely access to ongoing support and care^b^				
	*Breastfeeding‐groups are supported ± there is a parents' school ± breastfeeding café, mothers get contact options in case of breastfeeding problems after discharge* ^c^	58.3%	22.2%	63.2%	87.5%

^a^ German recommendations as given in (German Federal Institute for Risk Assessment 2007).

^b^ Recommendations as given in (WHO and UNICEF 2018).

^c^ Recommendations as applied in this study. Steps 1 and 2 were rated as “not implemented” if the head physicians reported not knowing the answer.

### 3.4 Interrelations Between the Social Structure and Breastfeeding Promotion

**FIGURE 3:**
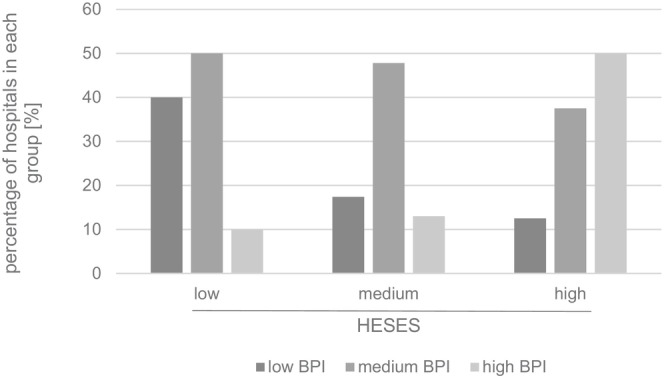
Breastfeeding promotion index (BPI) distribution within the HESES (hospital estimated socioeconomic status) groups. Kendall Tau c for correlation between HESES and categorized BPI = 0.287; *p* = 0.040.

HESES group differences regarding the number of implemented steps missed significance (low: 5.80 ± 1.32 (median 6.0) steps; medium: 6.17 ± 1.51 (median 6.5) steps; high: 7.00 ± 1.20 (median 7.5) steps; *p* = 0.66), whereas a higher HESES correlated significantly with a higher categorized (low, medium, high) BPI (*p* = 0.040; Kendall Tau c = 0.287; Figure 3). GISD group comparison revealed a similar result (in the low GISD group, 7.00 ± 1.55 (median 7.5) steps were implemented; medium GISD: 6.14 ± 1.28 (median 6.0) steps; high GISD: 6.00 ± 1.69 steps (median 6.0); *p* = 0.180). No significant correlation was detected between the GISD and the number of implemented steps per hospital (Spearman's Rho = 0.207; *p* = 0.227).

GISD differences between the BPI groups (low BPI: GISD 0.563 ± 0.234 (median 0.565); medium BPI: 0.424 ± 0.201 (median 0.449); high BPI: 0.386 ± 0.196 (median 0.415)) did not reach significance (*p* = 0.121).

### 3.5 Estimated Breastfeeding Rates at Discharge

The mean rate of any breastfeeding mothers at discharge as estimated by the maternity ward staff was 84.49% ± 9.41% (median 87.5%), while the estimated rate of exclusively breastfeeding mothers at discharge was 67.33% ± 21.45% (median 75.0%); for detail, see Table A2. Rates of any and exclusively breastfeeding mothers correlated significantly (Spearman's Rho = 0.773, *p* < 0.001).

**FIGURE 4:**
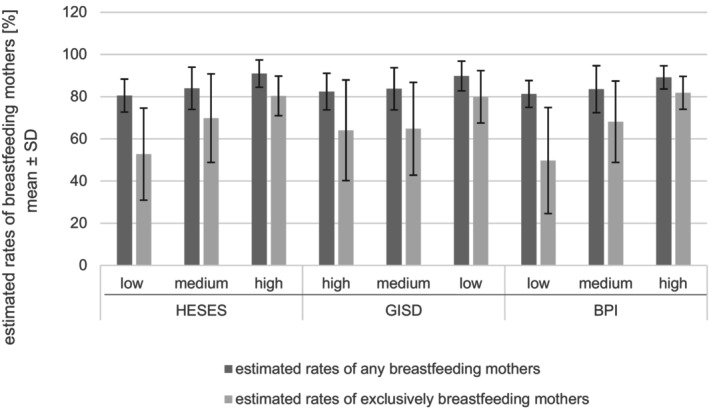
Group comparison of the estimated breastfeeding rates at discharge. Jonckheere‐Terpstra's test for each group comparison: HESES (hospital estimated socioeconomic status) groups: Any breastfeeding: *p* = 0.011* exclusive breastfeeding: *p* = 0.006**; GISD (German Index of Socioeconomic Deprivation) groups: Any breastfeeding: *p* = 0.088 (n.s.) exclusive breastfeeding: *p* = 0.139 (n.s.); BPI (Breastfeeding Promotion Index) groups: Any breastfeeding: *p* = 0.069 (n.s.) exclusive breastfeeding: *p* = 0.005**. Level of significance: *p < 0.5; **p < 0.01.

Comparison of the HESES groups revealed significantly higher rates of any and exclusive breastfeeding in hospitals with a higher HESES (any breastfeeding: *p* = 0.011; exclusive breastfeeding: *p* = 0.006; Figure 4). The GISD group comparison showed a similar non‐significant tendency (Figure 4). No significant correlation was found between the GISD and the estimated breastfeeding rates (any breastfeeding: *p* = 0.071; exclusive breastfeeding: *p* = 0.168).

In the BPI group comparison, any breastfeeding rates tended to increase with increasing BPI without reaching significance (*p* = 0.069). The estimated rates of exclusively breastfeeding mothers were significantly higher in the higher BPI groups (*p* = 0.005; Figure 4). This finding was confirmed by the significant correlation between the number of implemented steps and the estimated rate of any breastfeeding mothers (Spearman's Rho = 0.336, *p* = 0.045), as well as the estimated rate of exclusively breastfeeding mothers (Spearman's Rho = 0.525, *p* = 0.001).

In 38 hospitals (92.7%) services for outpatient support of families with a particular need for assistance were available. The proportion of hospitals providing such services tended to differ between the HESES groups (low: 100.0%, medium: 91.3%, high: 87.5%). Due to the small sample size and these minor group differences, this finding is reported descriptively without any significance testing. No clear trend was observable when comparing the GISD groups regarding the support service provision (high GISD: 100.0%, medium GISD: 88.0%, low GISD: 100.0%).

## 4 Discussion

This study revealed a high level of accordance between the HESES and the GISD. This finding suggests that head physicians are capable of estimating the prevailing social status in their hospitals' catchment areas. An interrelation between breastfeeding promotion in maternity hospitals and their socioeconomic background was identified. This interrelation was stronger using HESES than GISD as the socioeconomic measure. Breastfeeding rates at discharge differed significantly between the HESES groups, with a trend towards a higher HESES going along with higher breastfeeding rates. Therefore, HESES can serve as an indicator for increased breastfeeding promotion efforts needed in the corresponding maternity hospitals.

### 4.1 Assessment of the Socioeconomic Structure of the Hospital's Catchment Area

Since its original publication in 2017 (Kroll et al. 2017), the GISD has become an established measure for regional socioeconomic inequalities. There is evidence that the GISD can adequately represent the extent of socioeconomic deprivation of a region's population for public health studies. Due to the inclusion of analogous indicators, it is transferable to the socioeconomic status (SES; Michalski et al. 2022). This enables a comparison of the current findings from a regional or hospital‐related perspective with previous research conducted at the individual level.

The HESES groups differed regarding the median GISD significantly: a higher HESES was in line with a lower GISD. This suggests that head physicians have a realistic perception of the social structure of their hospitals' catchment areas. Though the validity of subjective estimations is generally limited, the two fundamentally different methodological approaches obviously measured congruently.

### 4.2 Breastfeeding Promotion, Information, and Support in Hospitals

The BPI based on the Ten Steps is an indicator for the in‐hospital breastfeeding promotion quality. There is evidence that breastfeeding outcome parameters (WHO and UNICEF 2018), such as exclusive breastfeeding rates (Hockamp et al. 2022), are improved by a high proportion of implemented steps. Furthermore, an improved implementation of the Ten Steps reduced racial disparities regarding breastfeeding in the US (Merewood et al. 2019). In contrast, the PROBIT study found a slight enlargement of social disparities after a breastfeeding promotion intervention based on the Ten Steps. Its impact for the interpretation of the current findings is unclear because data acquisition occurred in Belarus in 1996/1997 (Yang et al. 2014). The Ten Steps are suitable for the assessment of in‐hospital breastfeeding promotion quality, as well as for targeted improvement interventions (Pérez‐Escamilla et al. 2016).

On average, the hospitals implemented six of the Ten Steps, which corresponded to a medium BPI. The number of implemented steps was slightly lower and showed a smaller spread compared to the nationwide SuSe II study (Hockamp et al. 2022). Consequently, the 75th percentile as the cut‐off between a medium and a high BPI shifted. This results in a limited comparability.

### 4.3 Interrelations Between the Social Structure and Breastfeeding Promotion

This study revealed an interrelation between in‐hospital breastfeeding promotion and the socioeconomic structure of the hospitals' catchment areas. A higher HESES correlated significantly with a higher categorized BPI. GISD group comparison showed a non‐significant tendency towards a higher BPI in hospitals hosted by districts and independent cities with a low GISD. These results suggest that the districts and independent cities might not be the optimal surrogate for the hospitals' catchment areas. Therefore, HESES might better match the social structure of the hospitals' catchment areas. An alternative explanation can be that breastfeeding promotion in hospitals might be influenced by the perceived rather than the factual socioeconomic structure of the catchment areas.

While there is huge evidence for social disparities in breastfeeding habits, little is known about socioeconomic disparities regarding the provision of (in‐hospital) breastfeeding promotion, information, and support in Germany. Former studies which investigated individual mother‐infant pairs showed that breastfeeding is favored by a higher social status containing the mother's educational level (Kohlhuber et al. 2008; Lange et al. 2007; Libuda et al. 2014; Logan et al. 2016). This study indicates that breastfeeding promotion is weaker in hospitals located in regions with a more disadvantageous socioeconomic structure and vice versa. Therefore, a weak in‐hospital breastfeeding promotion might amplify the risk of poor breastfeeding in socioeconomically disadvantaged families. The impact of the socioeconomic status on breastfeeding might have been overestimated in the past due to this unknown confounder. On the other hand, breastfeeding promotion resources seem to be largely invested (by means of a high adherence level to breastfeeding promotion recommendations as the Ten Steps) in regions with a lower risk of unfavorable breastfeeding behavior. Considering the phenomenon of the inverse, respectively disproportionate, care law (Cookson et al. 2021), the current findings are not surprising. A Scottish qualitative study found that breastfeeding was not doubted as the optimum way of infant feeding by health professionals. Though, their views on breastfeeding promotion were ambivalent because of (i) concerns about a moralization of breastfeeding; (ii) unclear responsibilities regarding breastfeeding promotion in the health and educational system; and (iii) the perceived inefficacy of their work due to an overwhelming impact of family members and societal norms on the infant‐feeding decision (Marks and O'Connor 2015). The third aspect might partially explain the presence of the inverse care law in the current data: breastfeeding rates are known to be low in socially deprived populations (Hunt et al. 2021), and mothers of low social status are difficult to reach by support programs (Flothkötter et al. 2018). This might amplify the staff's pessimistic view on their influence and therefore reduce the motivation to invest time and effort in breastfeeding promotion, information, and support. Further research is needed to investigate a potential impact of the patient clientele's social structure on the staff's attitude in maternity hospitals. These aspects could be considered in tailored public health interventions and resource management in NRW.

### 4.4 Estimated Breastfeeding Rates

This study revealed higher estimated breastfeeding rates at discharge in hospitals situated in socially more advantaged regions (Figure 4 and Table A2). This is in line with previous research referring to the maternal socioeconomic status. In particular, maternal education is known to be a determinant of any and exclusive breastfeeding (Dubois and Girard 2003; Fernández‐Cañadas Morillo et al. 2017; Pitonyak et al. 2016). Whereas a higher educational level favors exclusive breastfeeding, a higher household income was identified as a risk factor for exclusive breastfeeding interruption in a Colombian study (Finnie et al. 2020). With regard to high‐income countries, data are inconclusive (Dubois and Girard 2003; Flacking et al. 2007). In the USA, structural and sociocultural factors have been identified that favor inequalities in breastfeeding and therefore enhance the risk of the cycle of malnutrition taking place even in a high‐income country. Due to the increase in these disparities in the course of climate change and crises such as the COVID‐19 pandemic (Tomori 2023), the need for targeted interventions becomes even more apparent.

We found higher rates of exclusive breastfeeding at discharge in the higher BPI groups and a significant correlation between the sum of implemented steps and the estimated breastfeeding rates. These findings are consistent with former research: it is known that in‐hospital breastfeeding promotion can have a supportive effect on breastfeeding (Pérez‐Escamilla et al. 2016), especially on exclusive breastfeeding (Hockamp et al. 2022). Our results are in line with a systematic review, which revealed a dose–response relationship between the extent of the implementation of the Ten Steps and breastfeeding outcome parameters (Pérez‐Escamilla et al. 2016).

The breastfeeding rates in our study were based on subjective estimations of maternity ward staff. Nevertheless, the high level of agreement with previous research as described above suggests a high significance of the present findings. The relationships between the breastfeeding rates and the BPI, as well as the HESES, suggest that the aforementioned interrelations between breastfeeding promotion and socioeconomic risk constellations might be of relevance from a public health perspective including the conception of pre‐ and postnatal support. Pediatricians can provide the latter, for example, by using preventive examinations in the first year of life for breastfeeding promotion (National Breastfeeding Committee n.d.).

The provision of outpatient support services seemed to be more likely in hospitals with a lower HESES; GISD group comparison revealed an unclear result. This suggests that the provision of support services might be influenced by the needs perceived by hospital staff, especially by head physicians.

### 4.5 Strengths and Limitations

The hospital‐based study design is advantageous because it avoids a selection bias caused by an overrepresentation of breastfeeding‐interested, native‐speaking, well‐educated mothers, which occurred in previous mother‐infant studies. As hospitals in NRW participated, this study provides specific data for this region, which could enable tailored public health interventions.

A limitation of the study is the small sample size of 36 and 41 hospitals, respectively. Aiming to provide information on the current state of breastfeeding promotion specifically for NRW, an enlargement of the catchment area was not conceivable. Further studies are necessary to confirm the results of this exploratory study. The hospital‐based study design required the inclusion of staff's subjective estimations (e.g., of the breastfeeding rates and self‐evaluation regarding the Ten Steps) into analyses because the demanded parameters are not routinely assessed by the hospitals. A nationwide standardized monitoring of breastfeeding and breastfeeding promotion would be the ideal concept but is not implemented in Germany yet. The interview questions were designed based on the German adaptation of the Ten Steps (German Federal Institute for Risk Assessment 2007), which could result in a slight reduction of the international comparability. However, the interview questions and the resulting data cannot fully reflect the WHO and UNICEF Ten Steps entirely. Detailed information on the Ten Steps as given by WHO and UNICEF, as well as by the German National Breastfeeding Committee, compared to the definitions used in this study are given in Table 1. Due to the great effort involved and the pandemic‐related visiting restrictions, on‐site verifications of the reported information by study staff were not possible. A social desirability bias can therefore not be excluded. The included hospitals were not perfectly representative for NRW regarding the annual birth rates and ownership. Regarding the perinatal care level, the study sample resembled the total of maternity hospitals in NRW. Due to the moderate participation rate, hospitals interested in breastfeeding may be overrepresented in terms of a self‐selection bias. Nevertheless, the observed relationships between the socioeconomic environment and breastfeeding might be transferable to German maternity hospitals in general. The GISD as a surrogate for the socioeconomic structure was obtained for the districts and independent cities hosting the hospitals. Though it can be assumed that these regions are largely representative for the hospitals' catchment areas, they do not fully correspond to them. Nine of the included districts and free cities hosted more than one of the surveyed hospitals. We assumed that the catchment areas of hospitals located closely together show a large overlap. The HESES probably depicts the catchment areas' social structure better. Therefore, the corresponding hospitals were not excluded from analysis.

## 5 Conclusion

The study revealed an interrelation between the quality of breastfeeding promotion (containing breastfeeding information and support) in maternity wards and their socioeconomic background. Breastfeeding rates and the BPI were related to the hospitals' socioeconomic environment: the lower the catchment area's predominant socioeconomic status, the lower were the breastfeeding rates at discharge and the weaker is the performed breastfeeding promotion. Both interrelations are stronger for the HESES than for the GISD. Therefore, the head physicians' estimations of the predominant socioeconomic status in the hospitals' catchment areas (HESES) might serve as an easily accessible marker for the hospitals' need for breastfeeding improvement interventions and the adequate allocation of resources. An improvement of the Ten Steps implementation rates can be one aspect of a required multi‐level approach for reducing social disparities and improving breastfeeding rates (Pérez‐Escamilla et al. 2023). This approach needs distinct strategies for reaching socially disadvantaged groups, such as peer counseling (Hunt et al. 2022).

Further studies are needed to confirm these new findings. The current data could also be considered in future research regarding the socioeconomic determinants of breastfeeding: an unfavorable breastfeeding behavior observed in families with a low social status might be affected by the lower level of breastfeeding promotion performed in socially disadvantaged regions as an example for the inverse care law (Cookson et al. 2021). Future research should also involve the subjective perceptions of the families concerned: a better understanding of the facilitators and barriers for breastfeeding and the utilization of healthcare services in underserved communities is crucial for the design of tailored interventions.

## Author Contributions

**Christina Kürten:** data curation (supporting), formal analysis (lead), visualization (lead), writing – original draft (lead). **Nele Hockamp:** data curation (lead), investigation (supporting), supervision (supporting), writing – review and editing (equal). **Kathrin Sinningen:** supervision (supporting), writing – review and editing (equal). **Erika Sievers:** conceptualization (supporting), writing – review and editing (equal). **Thomas Lücke:** funding acquisition (equal), supervision (lead), writing – review and editing (equal). **Mathilde Kersting:** conceptualization (lead), funding acquisition (equal), investigation (lead), supervision (supporting), writing – review and editing (equal).

## Conflicts of Interest

The authors declare no conflicts of interest.

## Supporting information


**Data S1:** Interview questions.

## Data Availability

The datasets used and analyzed during the current study are available from the corresponding author on reasonable request.

## Appendix A

**TABLE A1:** Hospital characteristics.

		Study sample			Federal state NRW
Ownership	Non‐profit	*n* = 24	58.5%		65.9%^b^
	Public	*n* = 14	34.1%		21.2%^b^
	Private	*n* = 3	7.3%		12.9%^b^
Involved in academic teaching	Yes	*n* = 38	92.7%		
	No	*n* = 3	7.3%		
Level of perinatal care	I	*n* = 20	48.8%	76.9%*	72.1%^c^
	II	*n* = 1	2.4%	4.8%*	10.2%^c^
	III	*n* = 5	12.2%	19.2%*	18.6%^c^
	IV	*n* = 15	36.6%		
Births in 2020	1.619 ± 727 (mean ± SD)				1198 ± 636^a^
Birth categories in 2020	≤ 500	*n* = 1	2.44%		8.6%^a^
	500–1000	*n* = 7	17.07%		37.7%^a^
	≥ 1000	*n* = 33	80.49%		53.6%^a^

^a^ Milupa Nutricia GmbH (2021).

^b^ German Federal Statistical Office (2022).

^c^ Richter et al. (2022).

* Percentages within the subgroup of perinatal care centers in the study sample (Level IV refers to obstetric departments without an associated pediatric department, thus no center of perinatal care).

**TABLE A2:** Estimated rates of any and exclusively breastfeeding mothers at discharge given as mean ± SD (median).

Group characteristics		Estimated rates of any breastfeeding mothers (%)	Estimated rates of exclusively breastfeeding mothers (%)
HESES	Low	80.5 ± 7.8 (80.0)	52.75 ± 21.81 (57.5)
	Medium	83.96 ± 10.02 (87.8)	69.82 ± 20.97 (80.0)
	High	90.94 ± 6.49 (92.0)	80.36 ± 9.38 (85.0)
GISD	High	82.39 ± 8.67 (80.0)	64.06 ± 23.85 (77.5)
	Medium	83.73 ± 10.01 (86.3)	64.78 ± 22.01 (65.0)
	Low	89.79 ± 7.02 (90.0)	79.93 ± 12.42 (87.5)
BPI	Low	81.28 ± 6.37 (80.0)	49.72 ± 25.14 (35.0)
	Medium	83.53 ± 11.12 (87.5)	68.11 ± 19.28 (72.5)
	High	89.13 ± 5.51 (90.0)	81.81 ± 7.8 (82.5)
Total		84.49 ± 9.41 (87.5)	67.33 ± 21.45 (75.0)
