# Supplementary material for: Breastfeeding Promotion in Maternity Wards From a Socioeconomic View
Source: Food Sci Nutr. 2025 Oct 15;13(10):e71045. doi: 10.1002/fsn3.71045 (PMC12528546; doi:10.1002/fsn3.71045)
Supplement: Supplementary file 1 — Data S1: Interview questions. [file FSN3-13-e71045-s001.pdf]

Supplementary material: Interview questions related to the data analysed in this manuscript

I) Questions posed to the head physicians

Introduction: We start the interview now. The first part is about the structural background to breastfeeding support in your hospital. We always start with the “baseline conditions” before the pandemic (2019), then any changes during the pandemic will follow.

1. Questions about the hospital in general

1.1 What kind of sponsor runs your hospital?
Instructions for the interviewer: Owners of the following categories can be named, query if necessary:
- Private ownership
- Ecclesiastical ownership
- Communal
- Federal state NRW
- Others

1.2 Is your clinic involved in academic teaching? (to be taken for granted in case of university hospitals)
- Yes
- No

1.3 Which level of perinatal care is provided by your clinic?
- Level I
- Level II
- Level III (obstetric department with associated paediatric clinic)
- Level IV (obstetric department without associated paediatric clinic)
- Others

1.4 How many births occurred in your clinic in the last year 2020?

2. Questions on the obstetric department

2.12 Does your clinic offer an integrative postnatal care concept (24h-rooming-in)?
- Yes
- No

3. Questions on financial challenges: None included in the presented study.

4. Questions on breastfeeding guidelines and breastfeeding information

4.1 Are there breastfeeding representatives in your clinic?
- Yes
- No

4.2 Were there breastfeeding promotion trainings for the hospital staff before the pandemic?
- Yes
- No

4.2.1 Which professional groups were trained?
Help by the interviewer: the following groups are conceivable:

- Physicians
- Nurses
- Paediatric nurses
- Midwifes
- Breastfeeding- and lactational consultants
- The whole team
- Others

4.3 What information offers on breastfeeding were available for pregnant women before the pandemic? (multiple responses possible)

- Information as part of an on-site antenatal class
- Information as part of an on-site breastfeeding preparation class
- Information in a medical/nursing consultation during pregnancy
- Information in a personal conversation during registration for birth
- Written information received during registration for birth
- Written information (on any occasion)
- Others

5. Questions on special needs

5.1 Which socioeconomic status prevails in the catchment area of your clinic with regard to breastfeeding promotion?

- High socio-economic status
- Medium socio-economic status
- High socio-economic status
- I do not know

5.2 Are there support services, e.g. advisory services, for families with special needs?

- Yes
- No

II) Questions posed to the maternity ward staff member with responsibility for breastfeeding

Introduction: Our interview focuses on the practical workflows regarding the breastfeeding management on your maternity ward. We always ask for the “baseline conditions” before the pandemic first. After that, changes within the pandemic may be surveyed.

1. Questions on breastfeeding information

We continue the topic breastfeeding information and focus on the information and counselling of the mothers delivering in your unit after discharge. Again, we talk about the prepandemic situation.

1.6 Which counselling services and support resources have you mentioned to mothers in case of breastfeeding problems at home? (this refers to precautionary recommendations/ nominations). Instruction for the interviewer: The following categories are conceivable as answers (multiple responses possible)

- Breastfeeding outpatient clinic in the maternity hospital
- Breastfeeding hotline in the maternity hospital
- Support at home by hospital staff
- Online counselling provided by the hospital
- Online counselling provided by others
- Addresses of freelance midwives
- Addresses of local breastfeeding groups
- Addresses of breastfeeding counsellors
- Other, namely:
- None

2. Questions on breastfeeding practices

2.2 When is a healthy newborn after vaginal delivery latched to the breast for the first time?

- Within one hour after birth
- Within two hours after birth
- Later
- I do not know

2.4 When is a healthy newborn after caesarean section latched to the breast for the first time?

- Within one hour after birth
- Within two hours after birth
- Later
- I do not know

2.5 Are mothers usually instructed during the first latch?

- Yes
- No

2.9 Several options are conceivable for the timing of breastfeeding in the course of the day. I will read out four options to you. You can give me feedback, which one of them applies best for your ward.

- Breastfeeding follows a fix schedule for all newborns
- Timing of breastfeeding depends on the needs of mother and child.
- The newborn should be breastfed at least eight times within 24 hours, but without a fix schedule
- The newborn should be breastfed at least ten times within 24 hours, but without a fix schedule
- Other approach:

<u>3. Questions on supplementary feeding</u>

3.2.1 Which procedure is performed usually (with regard to supplementary feeding of fluids to healthy breastfed newborns within the first three days of life)?

- Routine procedure (available for ward staff)
- Routine procedure (available for all mothers)
- Supplementary feeding only when specifically indicated
- I do not know
- Others

3.3 Now we focus on the feeding of formula to healthy breastfed newborns on your ward. Who decides about that? (multiple responses possible)

- Nurse in charge
- Midwife in charge
- Attending gynaecologist
- Attending paediatrician
- Mother
- I do not know
- Special regulation, that is:

3.5 How its supplementary feeding practiced if healthy breastfed newborns receive additional feeding? (multiple responses possible)

- By bottle
- By spoon/mug
- By fingerfeeding
- Breastfeeding-set
- Others:

<u>4. Questions on Not-Breastfeeding: None included in the presented study.</u>

<u>5. Questions on discharge</u>

5.1. How many mothers breastfed their child at discharge before the pandemic started? (Instruction for the interviewer: Indication of an order of magnitude is sufficient): %

5.1.1 How many mothers exclusively breastfed their child at discharge before the pandemic started, i.e. breastmilk only and no other fluids? (Instruction for the interviewer: Indication of an order of magnitude is sufficient): %

<u>6. Questions on visiting restrictions:</u> None included in the presented study.

<u>7. Questions on challenging aspects regarding in-hospital breastfeeding promotion from the ward staff's view:</u> None included in the presented study.
